# When to reveal what you feel: How emotions towards antagonistic out-group and third party audiences are expressed strategically

**DOI:** 10.1371/journal.pone.0202163

**Published:** 2018-09-07

**Authors:** Julia Sasse, Russell Spears, Ernestine H. Gordijn

**Affiliations:** 1 Max-Planck-Institute for Research on Collective Goods, Bonn, Germany; 2 Psychology Department, University of Groningen, Groningen, The Netherlands; Sapienza University of Rome, ITALY

## Abstract

In intergroup conflicts, expressed emotions influence how others see and react to those who express them. Here, we investigated whether this in turn implies that emotions may be expressed strategically. We tested whether emotion expression can differ from emotion experience, and whether emotion expression (more than emotion experience) is used to pursue specific goals. Specifically, we focused on whether support-seeking emotions (fear and sadness) are used to call for support from a powerful third party and contempt to distance from an antagonistic out-group. In two studies, using the same ostensible conflict, we manipulated whether participants communicated their emotions towards the out-group (no vs. yes) and third party (no vs. yes) and employed a between-subjects design in Study 1 (*N* = 86) and a within-subjects design in Study 2 (*N* = 83). In both studies, we found that members of a disadvantaged group expressed reduced support-seeking emotions towards the out-group than they experienced (i.e., in conditions without an audience), providing support for the assumption that emotion expression does not necessarily reflect experience. Further, in Study 2, we found in line with expectations that the goal to call for support was more important in the communication with the third party than with the antagonistic out-group. The goal was best predicted by expressed support-seeking emotions, providing support for the assumption that emotion expression is used to pursue goals. Interestingly, we only found this association for a beneficial goal (i.e., calling for support) and not for distancing, a destructive goal. These results support the proposed strategic use of emotion expression and as such advance our understanding of the function of expressed emotions.

## Introduction

Intergroup conflicts of any form, be it a minor dispute or a violent fight, come with a multitude of emotions, ranging from humiliation, fear, anger, and hatred to—in the best case—hope. Importantly, emotions are not only the product of conflicts but also affect conflicts [1]: Experienced emotions motivate actions [2,3] and expressed emotions shape reactions of others [4,5]. That expressed emotions seem to have the power to influence an audience raises the question whether emotions are expressed strategically, precisely because they trigger specific responses in an audience. In this paper, we set out to approach this question by investigating whether emotion expression may differ depending on an audience (i.e., antagonistic out-groups versus third parties) and whether the expression of specific emotions is associated with specific goals. By doing so we hope to advance our knowledge about the function of expressed emotions in intergroup conflicts.

### Emotions in intergroup conflicts

Anger, contempt, and fear are just some of the many emotions experienced during intergroup conflicts. These emotions do not arise out of the blue but depend on how members of a group evaluate their group’s position (with respect to status and power) and events related to the conflict (e.g., acts of offense or retaliation)–in other words how they appraise the situation [6,7]. In this paper, we focus on emotions commonly experienced by weak or disadvantaged groups as changing the status quo is of importance to them and thus strategy should be relevant.

Feeling weak or in a vulnerable position is associated with experiencing fear and sadness [6]. These emotions are also assumed to signal a sense of need [8]. Importantly, those appraisals not only precede emotion experience but audiences also seem to infer them from expressed emotions. Kamans and colleagues [4] showed that members of an uninvolved third party were more likely to support a disadvantaged group when its members expressed fear about their situation than when they expressed anger. This suggests that fear not only arises in response to feeling inferior but also enlists actions that may help to overcome the current situation. This is in line with van Kleef’s suggestion that (expressed) emotions constitute information that allows the audience to draw inferences about the cause of the emotion [9,10].

Anger is also an emotion that often arises during conflicts and it often has rather negative effects. In general, anger is more associated with powerful groups yet it also occurs in weaker groups in response to experienced injustice or unfair treatment [11]. Interestingly, while Kamans and colleagues found that disadvantaged groups should not express anger about the perpetrator out-group towards a third party [4], de Vos and colleagues in fact found it can have positive effects if they express it to the perpetrator out-group themselves [5], suggesting that the effects of expressed anger can be manifold. Specifically, they showed that perceiving a group as angry can actually increase empathy for this group, which in turn motivates more constructive action intentions. The reason for this, they argue, is that by showing anger the group communicates that it has been treated in an unfair way. This means anger not only arises in response to experienced injustice but it also seems to communicate it (at least under certain conditions). De Vos and colleagues [5] compared the effects of pure anger with anger mixed with contempt and showed that the latter combination has rather detrimental effects as it leads groups that are confronted with this mixture of anger and contempt to react destructively. This is in line with Fischer and Roseman’s [12] characterization of contempt as an emotion that arises when after a relationship has been harmed repeatedly and distance rather than reconciliation is sought.

To summarize, fear, anger, and contempt are emotions likely to be experienced by disadvantaged group during conflict yet their expression leads to very different reactions from audiences. Based on the findings described above disadvantaged groups should choose to express fear (and sadness) if their goal is to enlist third party support. An out-group’s willingness to work constructively on the other hand seems to be positively influenced by expressed anger while contempt should only be openly expressed if the goal is to end a relationship. Although people are unlikely to be fully aware of these specific influences of emotion expression, their lay-theories about how it could help them to reach specific goals might lead them to express emotions strategically.

### Shaping emotion expression strategically

As expressed emotions pose information for an audience they may be regarded a channel of communication with an audience. Undoubtedly other channels of communication are given such as language (i.e., verbalization of goals) and actions but we consider emotion expression of special importance for several reasons. Firstly, emotion expression is subtler than language and actions, and though it may lead to negative reactions it is not obviously punishable or costly. This notion of subtlety may further be strengthened by the seemingly common idea that being emotional is authentic and contrary to being rational (or indeed strategic), which makes the deniability of any attempt to influence more plausible than for language or actions. More importantly, emotions may be efficient as they convey powerful information for the audience [9] but at the same time capitalize on a certain ambiguity. They communicate a message without making it explicit or appearing deliberate and have a “plausible deniability” less possible in overt speech (“weakness as strength”). Emotions may thus incur few costs in terms of both effort and potential sanctions. Lastly, research suggests that the opportunity to express (negative) emotions in response to an unfair offer reduces people’s inclination to punish [13]. This further supports the idea of a general understanding of the communicative function of expressed emotions and even suggests that expressing emotions may be seen as a signal as strong as an action (such as punishment).

Using emotion expression as a communicative tool presupposes that emotions can–at least to a certain extent–be manipulated by the expresser. Indeed, research has shown that emotions can be influenced (i.e., regulated) intentionally and this is not only done in order to feel more positive emotions but also negative emotions if this is considered beneficial (e.g., experiencing anger in preparation for a confrontation [14,15]. Such instrumental emotion regulation has been investigated in the context of interpersonal emotions but also occurs for group-based emotions [16]. While emotion regulation shows the general malleability of emotions, research has strongly focused on the regulation of experienced emotions and its consequences for the individual (or in-group). The instrumentality of emotion regulation should however not be limited to experienced but also expressed emotions. Evidence that emotions are intentionally expressed (or suppressed) stems to a large part from research showing how emotions may be expressed in accordance with culture-specific norms and following display rules [17,18, 19] but this tells us little about whether and how specific goals are pursued. Some initial evidence for this was provided by Andrade and Ho [20] who exposed participants to an unfair treatment to provoke anger. This anger was expressed to a greater extent to the opponent than it was reported confidentially. Importantly with respect to whether emotion expression is goal-directed, participants were aware of the fact that they changed their emotion expression and did so to obtain a fairer offer subsequently.

### The present research

In this paper, we are interested in whether emotions are expressed strategically in the context of a group facing a potential collective disadvantage. The notion of strategy entails two important components: The basic first component is the assumption that emotion expression may differ from emotion experience and that expression about the same subject may differ from audience to audience. That allows emotion expression to be tailored to specific goals rather than being fully driven by experience. Naturally, we expect emotion experience and expression to correlate, yet an emotion can be played up or down when it comes to expression.

As the second and necessary component of strategic expression, we propose that emotions are used to pursue specific goals. As such, we should be able to find a direct association between expressed–over and above experienced–emotions and such goals. While the first component is necessary for allowing strategic tailoring of emotion expression in principle, it does not necessarily have to manifest in observable (or rather measurable) differences between experience and expression: It may happen that experience and strategic expression correspond. However the association between expression and goals should always be detectable.

In Study 1, we tested the basic first component of strategic emotion expression that emotion expression may differ from experience. To do this, we investigated how members of a disadvantaged group experience emotions about their situation and express it towards an antagonistic out-group (that is, the group which is responsible for the disadvantaged situation) and a third party (that is, a group which is not responsible for the disadvantages but may potentially help to overcome them). Following from the findings regarding the effects expressed emotions have on third parties and out-groups [4,5] we expect members of a disadvantaged group to express more support-seeking emotions than they experience towards a third party and to express more contempt towards the out-group in response to their offense. As the results for the effects of anger have been mixed we explore its strategic use exploratively. Potentially, anger is used to stress experienced disadvantage yet it may also be reduced given its reputation (albeit not always warranted in reality) as a destructive emotion. In Study 2, we further extend the exploration of strategic emotion expression and test whether the association between expressed emotions and goals is indeed stronger than between experienced emotions and goals, which is the second component of strategic emotion expression. Specifically, we expect that the goal of expressing support-seeking emotions is to enlist support, and that of contempt is to distance from the out-group, based on the effects that these emotions have on audiences [4,5].

We tested our predictions in a manufactured conflict, which gave us full control over the properties of the conflict. It may for example be that the extent to which a third party or the out-group have (perceived) control over the outcome of the conflict influence both support-seeking emotions and contempt. To control for this, we assigned all power to the third party which should stimulate intentions to win its support and at the same time to distance the in-group from the out-group. While we used the same conflict in both studies we used different experimental designs to measure emotion experience and expression to control for methodological limitations. To make it more credible that participants were actually communicating with an audience we employed a between-subjects design in Study 1. In Study 2 we measured emotion experience and expression towards different audiences repeatedly to stress potential differences and employed bogus physiological measures to detect potential experimenter effects and potential diminution in repeated emotion reports.

## Study 1

### Method

#### Participants and design

International (i.e., non-Dutch) undergraduate students participated in the study and either received course credit or could enter a lottery (four 25-euro Amazon-vouchers). We excluded 28 participants who did not finish the study and two participants who indicated that one of their parents was Dutch (per condition, numbers of excluded participants and of those that dropped out after the introduction of the manipulation amounted to two to three and were thus comparable across conditions, final sample *N* = 86, age: *M* = 21.41, *SD* = 2.05; gender: 42 female, 13 missing values).

The study was approved by the Psychology Ethical Committee of our host institution, and conducted in accordance with its ethical guidelines. Upon accessing the study participants were informed about its format, duration, reward, and anonymity. They were asked to give consent to participate by moving forward in the online questionnaire. At the end of the study participants were fully debriefed and thanked for their participation.

Participants were randomly assigned to conditions in a 2 (emotion expression towards out-group: no vs. yes) by 2 (emotion expression towards third party: no vs. yes) between-subjects design. The combination of these factors resulted in a condition without any audience (“no audience condition”) where participants reported how they experienced their emotions confidentially and three emotion expression conditions where participants got to communicate their emotions to either single audiences (i.e., only the out-group or the third party) or both audiences at the same time. As main dependent variables we assessed anger, contempt, and support-seeking emotions.

#### Materials and procedure

We conducted the study online (using Qualtrics) and consent was obtained from all participations. To obscure the actual aim of the study we presented it as a survey about studying abroad to get insight into international students’ life and their experiences. Participants received a full debriefing at the end of the study.

The first part of the study focused on the experiences of international students to make the social identity of international students in relation to Dutch students salient: We assessed participants’ identification with international students [21], and we asked participants to rate seven statements about their experiences with Dutch and international students (e.g., “I experience Dutch students to be friendly and cooperative” or “I prefer to stay amongst students from my home country”).

Next, we introduced a fake conflict: Participants received information about a new law enabling universities to raise tuition fees individually due to the financial crisis. Based on this law a group of Dutch students (antagonistic out-group) wrote a proposal for higher tuition fees of 3000 euros per year solely for international students (i.e., participants’ in-group). The proposal was justified by the claim that international students profit from the Dutch education system but do not contribute to society (e.g., by paying taxes). A University Committee, consisting of staff members, would decide about the proposal and either accept or reject it and thus served as a (powerful) third party in this conflict (note that we described the group of staff members as diverse, with a large number of international employees in order to avoid (perceived) overlap between out-group and third party). Importantly, other than the antagonistic out-group this third party was not responsible for the in-group’s disadvantaged situation but was potentially able to help to overcome it, which served to qualify it as a source for support. Subsequently, participants were asked how they appraised the proposal and how they felt about it. Before giving their answers, the audience manipulation was introduced by informing participants that the results of this survey would either be confidential (i.e., no audience condition; reflecting emotion experience), communicated to Dutch students (out-group audience condition), to the University Committee (third party audience condition) or to both groups (both audiences condition).

First, participants appraised the proposal with regard to injustice, morality, uncertainty, expectancy, and sense of controllability. We expected that the proposal should be appraised as unjust, immoral and causing uncertainty and to a certain extent as expected, irrespective of the audience. Controllability should be low as international students did not have a say in the decision making process. Each appraisal was assessed with four items [with two being reversed coded; 7-point Likert scale, 1 = strongly disagree, 7 = strongly agree; examples: injustice “The proposal is unjust” (α = .80) [22], morality “The proposal is immoral” (α = .79), uncertainty “The proposal renders me uncertain about my future” (α = .83), expectancy “The proposal was to be expected” (α = .87), uncontrollability “The proposal is beyond our control” (α = .79)].

Participants were then asked to report anger (angry, irritated, revolted, Cronbach’s α = .84), contempt (contemptuous, disdainful, scornful, α = .81), and support-seeking emotions, which included items covering sadness and fear (sadness: sad, depressed, down, α = .80; fear: scared, anxious, frightened, α = .93) on 7-point scales (1 = none, 7 = a lot). The reliability of all fear and sadness items together was very high (α = .91) and supports our assumption that–in the given context—they serve the same central function (i.e., support-seeking), thus we combined them to support-seeking emotions.

Along with these focal dependent measures, relevant for testing our hypotheses, we assessed additional measures exploratively, such as perceived likelihood of influence and procedural fairness. Basic results for those measures are not reported here but provided in S1 File and S1 Table.

### Results

We first checked the distribution of all subsequently reported dependent measures by inspecting skewness and kurtosis; the z-transformed results are reported in Table 1. We applied a criterion of 1.96 (*p* < .05) and results suggest that data were distributed normally.

**Table 1:** Z-scores of skewness and kurtosis separately per audience conditions in Study 1.

**A Skewness**				
	Out-group audience			
	No		Yes	
	Third party audience		Third party audience	
	No	Yes	No	Yes
Support-seeking emotions	-0.11	0.00	0.32	-0.83
Contempt	0.84	-0.70	0.31	0.62
Anger	-0.23	-1.36	0.18	-0.62
Identification	-1.99	-1.05	-0.28	-0.17
Injustice	1.20	-1.18	0.38	-0.68
Morality	0.97	-1.40	-0.11	-0.26
Uncertainty	1.22	0.17	0.82	-0.30
Expectancy	0.92	0.10	1.03	-0.40
Controllability	-1.14	-0.17	-0.53	0.23
**B Kurtosis**				
	Out-group audience			
	No		Yes	
	Third party audience		Third party audience	
	No	Yes	No	Yes
Support-seeking emotions	1.42	0.03	-1.56	-0.61
Contempt	1.31	-1.04	0.66	0.89
Anger	0.08	-0.63	-1.24	-1.05
Identification	1.91	0.82	-1.17	-0.03
Injustice	-0.52	0.52	-1.12	-0.16
Morality	-0.04	1.92	-0.87	-1.35
Uncertainty	-0.64	0.28	-0.70	0.23
Expectancy	-0.63	-0.94	-0.68	-1.08
Controllability	0.62	-1.46	-0.28	-0.36

We then subjected all dependent measures to separate 2 (emotion expression towards out-group audience: no vs. yes) x 2 (emotion expression towards third party audience: no vs. yes) between subjects ANOVAs. First, we report participants’ identification with international students and how they appraised the situation to ensure that we successfully introduced an intergroup conflict in which participants are members of the disadvantaged group.

#### Identification

As expected, identification was on average moderate (*M* = 4.44, *SD* = 0.83) and did not differ between conditions, *p*s ≥ .15 (analyses with identification as a moderator are reported in S2 File as they are not central to the current story).

#### Appraisals

How participants appraised the proposal did not differ depending on audience, *p*s ≥ .17. Comparisons of means to the scale midpoint (across conditions) showed that, overall, the cover story created the intended perception of mistreatment amongst participants (see Table 2).

**Table 2:** Means and standard deviations (in parentheses) of appraisals in Study 1 and Study 2.

	Injustice	Immorality	Expectancy	Uncertainty	Controllability
Study 1	4.98***	4.79***	4.61***	4.55***	4.04
	(1.13)	(1.06)	(1.20)	(1.18)	(1.03)
Study 2	4.46***	4.23^+^	4.2	4.17	4.15
	(1.15)	(1.10)	(1.15)	(0.97)	(1.13)

*Note*. In Study 2, means are reported across bogus pipeline conditions. Asterisks indicate differences from scale midpoint (4).

^+^*p* < .1

*p < .05

**p < .01

***p < .001.

#### Emotions

**Fig 1:**
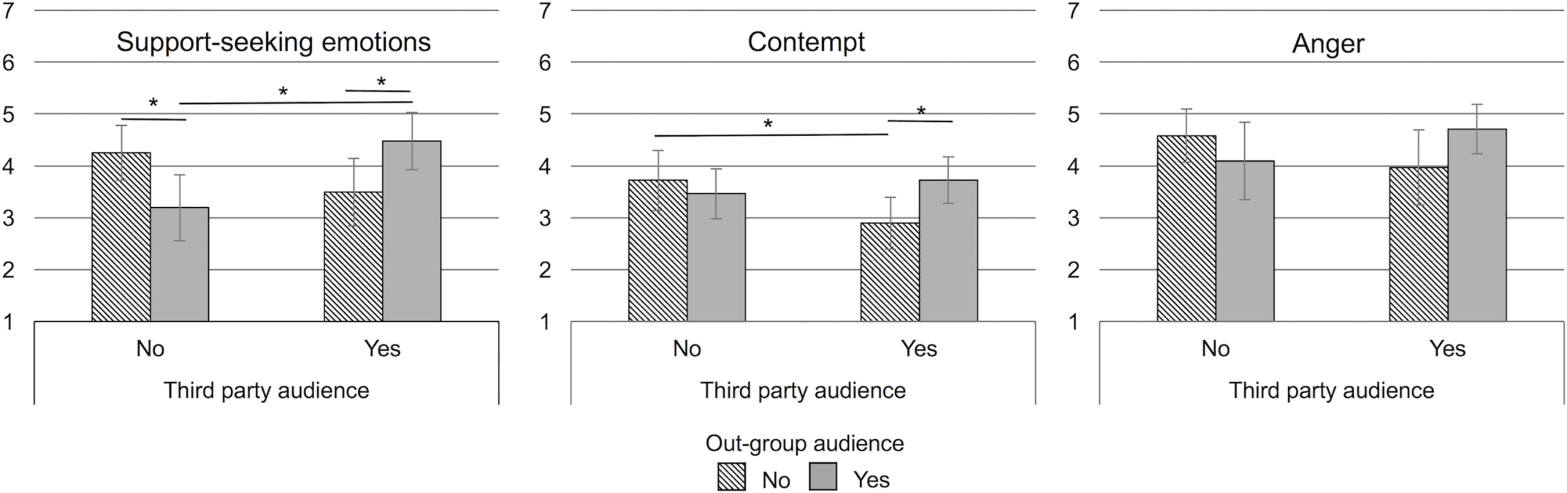
Support-seeking emotions, contempt, and, anger as experienced and expressed towards different audiences in Study 1. Error bars depict 95% confidence intervals. * *p* < .05.

We subjected support-seeking emotions, anger, and contempt to separate 2 (emotion expression towards out-group audience: no vs. yes) x 2 (emotion expression towards third party audience: no vs. yes) between subjects ANOVAs. Comparisons between the no audience condition (i.e., reflecting emotion experience) and single audiences (i.e., out-group or third party) were relevant to answer the question whether expression towards different audiences differs from expression (the both audiences condition completed the experimental design and may provide insight into which audience determined emotion expression when both audiences were addressed). Thus, if we obtained significant interactions between the out-group audience and third party audience factors we computed simple main effects to test whether single audiences differ from the no audience condition. Results for each emotion are depicted in Fig 1.

Neither out-group audience nor third party audience showed a main effect on support-seeking emotions, *p*s > .36, however, the interaction was significant, *F*(1,82) = 12.71, *p* = .001, η_p_^2^ = .13. Participants expressed support-seeking emotions less towards the out-group than they reported experiencing them (no audience condition), *F*(1,82) = 6.52, *p* = .01, η_p_^2^ = .07. The expression towards the third party was marginally reduced, *F*(1,81) = 3.41, *p* = .07, η_p_^2^ = .04. The expression towards both audiences together was higher than towards the out-group, *F*(1,82) = 6.19, *p* = .02, η_p_^2^ = .07, and towards the third party, *F*(1,82) = 10.32, *p* = .002, η_p_^2^ = .11.

Neither factor showed main effects on contempt, *p*s ≥ .24, but the interaction was significant, *F*(1,82) = 5.10, *p* = .03, η_p_^2^ = .06. The expression of contempt towards the third party was lower than experience, *F*(1,82) = 5.84, *p* = .02, η_p_^2^ = .07. In contrast, communication of contempt towards the out-group did not differ from the no audience condition, *F*(1,82) = 0.54, *p* = .46, η_p_^2^ = .01. Also compared to both audiences together expression towards the third party was reduced, *F*(1,82) = 6.24, *p* = .01, η_p_^2^ = .07, while expression towards the out-group did not differ, *F*(1,82) = 0.62, *p* = .43, η_p_^2^ = .01.

Neither out-group audience nor third party audience showed a main effect on anger, *p*s ≥ .68. The interaction was significant, *F* (1,82) = 4.16, *p* = .045, η_p_^2^ = .05, yet none of the simple effect tests yielded significance (*p*s ≥ .15). Only the expression towards the third party was marginally lower than towards both audiences together, *F*(1,82) = 3.17, *p* = .08, η_p_^2^ = .04.

### Discussion

We tested the first component of strategic emotion expression, namely that expression may differ from experience. In the present context, we expected the expression of support-seeking emotions to be more important in the communication with a third party. Interestingly, we did not find the expected increase in the expression towards the third party but rather a decrease in the communication with the out-group. The expression towards the third party on the other hand rather resembled experience in the no audience condition despite being somewhat lower as well, albeit not significantly so (and this was an unpredicted tendency so should thus be interpreted cautiously). These results support the general hypothesis that expression may differ from experience, though it manifested in a reduction of support-seeking emotions towards the out-group rather than an increase towards the third party (so the relative relation between these audiences is as expected). Possibly, in-group members were less willing to admit their weakness and tried to play down their need for support when communicating towards the out-group. Such an admission might represent a loss of face, and as very little support can be expected from the out-group it would be perceived as damaging the in-group’s image. At the same time, we found that contempt expression towards the third party was lower than experience: Presumably, it is not desirable to express destructive emotions to a third party but to appear reasonable and cooperative. Anger expression did not differ from experience or between audiences. This does not support the idea that anger (unlike contempt) may be reduced to avoid potentially destructive responses from the out-group. Rather, it may indeed be expressed to communicate the experience of wrongdoing.

Comparisons between single audiences and both audiences together were less clear but it is noticeable that expression towards both audiences was generally high. Thus, if participants reduced their support-seeking expression (towards the out-group) or contempt expression (towards the third party) strategically this strategy does not seem to be applied when both audiences were present at the same time. It rather seems as if the respective other audience overrode the reducing the effect. In other words, if participants were willing to express (somewhat) more support-seeking emotions towards the third party and contempt towards the out-group they did so even if the respective other audience was addressed as well. A tentative conclusion could be that anticipated positive outcomes from expression overrode anticipated negative outcomes and thus led to expression similar to experience.

In summary, Study 1 supported the prediction that emotion expression may differ from experience and showed that differences are not general but specific to different audiences yet we can thus far only speculate about the reasons for this. Therefore in Study 2 we turned to investigating goals associated with emotion expression.

## Study 2

While Study 1 provided initial support for the first component of strategic emotion expression, namely that emotion expression may differ from experience, Study 2 focused on strategic considerations presumed to underlie differences in emotion experience and expression by investigating the role of emotion expression in goal pursuit. In the presented context, strategic consideration should be most important for the expression towards the third party, which was presented as holding the power of decision, and thus was the audience that can actually improve the in-group’s situation. We thus predicted that the goal of members of the disadvantaged group would be to seek support from the third party, and that expressed help seeking emotions would be used to try to achieve this goal. With respect to the out-group on the other hand we predicted that the need for support would not be disclosed but the main goal would be to create distance: The out-group was responsible for the proposal creating injustice and at the same time did not have any influence on the further decision making process. We expected that expressed contempt would be used to try to achieve this goal.

We also aimed to make the difference between experienced and expressed emotions more salient. To do this we asked participants firstly how they feel about the conflict and secondly how they would like to express their emotions towards each of the audiences in a repeated measures design. To reduce the influence of repeated assessment and to increase confidence in our measurements of experienced and expressed emotions we further employed two different bogus physiological measures [23].

### Method

#### Participants and design

International undergraduate psychology students participated in this study in exchange for course credits. Data from two participants had to be excluded because they knew about the cover story or partly grew up in the Netherlands (final sample *N* = 83, age *M* = 20.77, *SD* = 2.34, 65 female).

The achieved power in Study 1 was rather low (e.g., interaction effect on contempt .64) so to increase power in Study 2 we computed the required sample size with G-Power [24]. Using audience this time as a within-subjects factor and based on the effect size for contempt in Study 1 (*f* = .25; as a more conservative benchmark compared to the effect size for support-seeking emotions), α = .05, power = .80, and (expected) correlation between the measures *r* = .70 a sample size of 15 would be required. In addition to replicating the effect of audience we further expected that emotion reports should not be influenced by either of the bogus pipeline manipulations. If however either of the bogus pipeline factors would show a small interaction effect with audience (*f* = .10) a sample size of 84 would be required to detect it (α = .05, power = .80, and (expected) correlation between the measures *r* = .70) Our sample size should thus be sufficient to detect such an effect.

The study was approved by the Psychology Ethical Committee of our host institution, and conducted in accordance with its ethical guidelines. Upon arrival to the lab participants were informed about its format, duration, reward, and anonymity, and asked to give written consent to participate. In conditions in which we used bogus physiological measures participants were informed that those measures we neither dangerous nor invasive in any way. At the end of the participation participants were fully debriefed and thanked for their participation.

We used a 2 (experienced emotions: bogus pipeline on vs. off) x 2 (expressed emotions: bogus pipeline on vs. off) x 2 (emotion expression towards out-group audience: no vs. yes) x 2 (emotion expression towards third party audience: no vs. yes) mixed design, with the latter two factors (i.e., audiences) being within-subjects factors. Participants were randomly assigned to the bogus pipeline conditions. Again, anger, contempt, and support-seeking emotions were the main dependent variables. In addition, we assessed goals of emotion expression.

#### Materials and procedure

We used the same cover story and conflict as in Study 1. Participation took place in the lab in individual cubicles and participants in conditions including one of the bogus pipeline procedures received additional information about physiological measures and that these were neither invasive nor dangerous. The order of premeasures and dependent variables as well as the cover story were similar to Study 1. To keep the study duration reasonable we used a single item measure of identification [25].

Phase 1 was designed to assess emotion experience (i.e., no audience presented). For half of the participants we used “facial response sensors”, four electrodes attached next to and below both eyes and connected to an amplifier. These were ostensibly able to detect activity patterns in facial muscles from which the experience of distinct emotions can be inferred. Allegedly, these muscular responses are not controllable and thus a mismatch between muscular activity and emotion reports would reveal insincerity.

Participants were then asked how they feel about the proposal. As the reliability of emotion scales in Study 1 was very high and we aimed to keep the study duration reasonable (taking into account the repeated measures design) we excluded the adjectives that reduced reliability the least [correlations for all repeated measurement points, *p*s. ≤ .001: anger (angry, irritated, *r*s > .56), contempt (scornful, disdainful, *r*s > .36), fear (scared, frightened, *r* > .82) and sadness (depressed, down, *r*s > .56), combined support-seeking emotion measures αs > .88].

In Phase 2, we assessed emotion expression towards different audiences repeatedly. Here, the second bogus pipeline procedure was used to investigate whether emotion expression was reported sincerely, i.e., as emotions actually would be expressed towards each particular audience. Half of the participants were assigned to this second bogus pipeline physiological measurement. For these participants, a single electrode, introduced as “deviation polygraph”, was attached to their left hands at the beginning of the study. This electrode was ostensibly able to detect changes in skin conductance response. Such changes were stated to detect increased arousal and thus indicative of an attempt to conceal one’s actual expression intentions.

Phase 2 began with the assessment of how participants would express their emotions towards the out-group (using the same emotion adjectives as described above). After this we measured different goals of emotion expression (7-point scale, 1 = strongly disagree, 7 = strongly agree). First, to measure the goal to seek support we used two items and later on computed the mean of the responses (“My intention is to show that we need assistance”, “My intention is to show that we are victims”, correlations for all audiences *r*s ≥ .33, *p*s < .05). Second, to measure distancing from the out-group participants were asked to rate the extent to which they agreed with the following statement “My intention is to show that our relationship with Dutch students is disrupted”. We embedded the items for these two focal goals in a list of several items.

Next, all measures were repeated with the only difference that participants were asked to imagine that they were addressing the third party. In a third round participants were asked to respond as if both audiences were present at the same time.

Although the two bogus physiological measures seem to be similar, they addressed two different issues: In Phase 1, facial response sensors were supposed to ensure that participants report how they truly feel about the proposal. On the contrary, the deviation polygraph in Phase 2 was intended to make participants express their emotions like they would when actually facing the respective audience. This procedure helped to overcome shortcomings of the experimental setting: Reporting emotions repeatedly may be influenced by consistency concerns, thus producing similar emotion reports in each condition while suppressing existing strategic considerations. In addition, when reporting emotions four times, a decline in levels of emotions may be expected. The constant reminder of the necessity to be sincere should prevent this.

### Results

We checked the distribution of all subsequently reported dependent measures by inspecting skewness and kurtosis; the z-transformed results are reported in Table 3. Applying a criterion of 1.96 (*p* < .05), there was little reason for concern for the central measures of emotions and goals while the results for identification indicated a build-up of high scores and a heavy-tailed distribution in the conditions in which only one bogus pipeline procedure was used.

**Table 3:** Z-scores of skewness and kurtosis separately per bogus pipeline conditions in Study 2.

**A Skewness**								
		Experienced emotions bogus pipeline						
		No				Yes		
		Expressed emotions bogus pipeline				Expressed emotions bogus pipeline		
		No		Yes		No		Yes
Support-seeking emotions no audience		-0.82		0.37		0.15		0.91
Support-seeking emotions out-group		0.61		0.60		1.71		1.21
Support-seeking emotions third party		-0.13		0.13		1.49		0.70
Support-seeking emotions both audiences		0.46		0.59		1.97*		0.31
Anger no audience		-0.81		0.75		-1.41		-0.85
Anger out-group audience		0.51		0.42		-0.18		-0.78
Anger third party audience		1.05		-0.29		-0.14		-0.97
Anger both audienes		0.92		0.09		0.18		0.27
Contempt no audience		-1.94		-0.90		-0.38		0.23
Contempt out-group audience		-1.11		0.46		-1.73		-0.62
Contempt third party audience		-0.97		-0.61		-1.01		-1.08
Contempt both audience		-0.43		-1.29		-0.83		-1.05
Need for support out-group audience		-0.12		-0.04		0.05		0.89
Need for support third party audience		-0.99		-0.98		0.04		0.19
Need for support both audiences		0.10		-0.61		0.03		0.11
Distancing out-group audience		1.52		1.66		2.07*		-0.58
Distancing third party audience		-0.34		1.02		0.47		0.09
Distancing both audiences		-0.42		1.32		0.77		-0.11
Identification		-1.19		-4.25*		-2.84*		-0.91
Injustice		-0.73		0.93		-0.40		-0.25
Morality		0.62		0.64		0.31		-1.39
Uncertainty		-0.73		0.53		1.17		-0.02
Controlability		-1.79		-0.55		0.13		0.62
Expectancy		-0.19		-0.03		0.05		0.23
**B Kurtosis**								
		Experienced emotions bogus pipeline						
		No			Yes			
		Expressed emotions bogus pipeline			Expressed emotions bogus pipeline			
		No	Yes		No		Yes	
Support-seeking emotions no audience		-1.02	-1.31		-0.94		-0.42	
Support-seeking emotions out-group		-0.86	-0.91		0.13		-0.14	
Support-seeking emotions third party		-1.42	-1.18		-0.53		0.06	
Support-seeking emotions both audiences		-1.14	-1.15		0.00		-0.16	
Anger no audience		-1.21	-1.26		0.94		-0.88	
Anger out-group audience		-1.24	-0.56		-0.66		-1.27	
Anger third party audience		-0.61	-1.02		-1.18		-0.28	
Anger both audienes		-0.71	-0.49		-0.36		-1.37	
Contempt no audience		0.62	0.15		-0.12		0.20	
Contempt out-group audience		-1.43	1.97*		-0.52		-0.87	
Contempt third party audience		-1.40	-0.11		-0.74		-0.48	
Contempt both audience		-1.36	-0.73		-0.83		-0.63	
Need for support out-group audience		0.05	-0.08		-0.44		-0.77	
Need for support third party audience		-0.16	-0.51		-0.09		-0.90	
Need for support both audiences		-0.58	0.37		1.57		-0.98	
Distancing out-group audience		-0.11	0.51		0.37		-0.83	
Distancing third party audience		-0.39	-0.75		-1.51		-0.95	
Distancing both audiences		-1.24	-0.66		-0.85		-0.78	
Identification		0.13	7.07*		2.95*		-0.40	
Injustice		0.09	-0.53		-0.68		-0.93	
Morality		-0.93	-0.43		-0.34		-0.27	
Uncertainty		-0.81	-0.98		-0.23		0.09	
Controlability		0.34	0.65		-0.31		-0.22	
Expectancy		-1.04	-0.85		-0.90		-0.26	

First, we report the results for identification and appraisals. During the assessment of these measures none of the two bogus pipeline decides was “active”, but were already attached in the respective conditions. To ensure that identification and appraisals were not influenced by this, we computed separate 2 (experienced emotions bogus pipeline: no vs. yes) x 2 (expressed emotions bogus pipeline: no vs. yes) between subjects ANOVAs, not expecting any differences between conditions.

#### Identification

Identification among participants was high (*M* = 5.71, *SD* = 1.01) and comparable across the four bogus pipeline conditions, *p*s > .27.

#### Appraisals

As expected, the mere presence of electrodes had no effects on appraisals, *p*s ≥ .22. Further, as we had intended, comparisons to scale midpoints showed that participants appraised the proposal mainly as unjust and somewhat immoral (Table 2).

#### Emotions

Separate for each emotion we computed a 2 (experienced emotions bogus pipeline: no vs. yes) 2 x (expressed emotions bogus pipeline: no vs. yes) x 2 (expression towards out-group audience: no vs. yes) x 2 (expression towards third party audience: no vs. yes) mixed ANOVA. We computed simple main effects to follow up on significant interaction effects.

The two bogus pipeline procedures had very little influence on emotion reports, *p*s ≥ .08. Only for anger did we find a significant interaction between expressed emotions bogus pipeline procedure and third party audience, *F*(1,79) = 4.64, *p* = .03, η_p_^2^ = .06. We computed simple main effects and found that the crucial comparisons, i.e., anger reports in the presence or absence of the bogus electrodes, were not significant (no third party audience, *F*(1,79) = 0.02, *p* = .88, η_p_^2^ < .001; with third party audience, *F*(1,79) = 1.56, *p* = .22, η_p_^2^ = .02). The fact that participants in conditions without bogus pipeline measures reported their emotions similarly to participants in bogus pipeline conditions (in which insincere reports would be unmasked) increases our confidence in the self-reports of emotions (as used in Study 1). Thus, we can assume that emotions were communicated as experienced (Phase 1) and as they would be expressed to the audiences (Phase 2).

**Fig 2:**
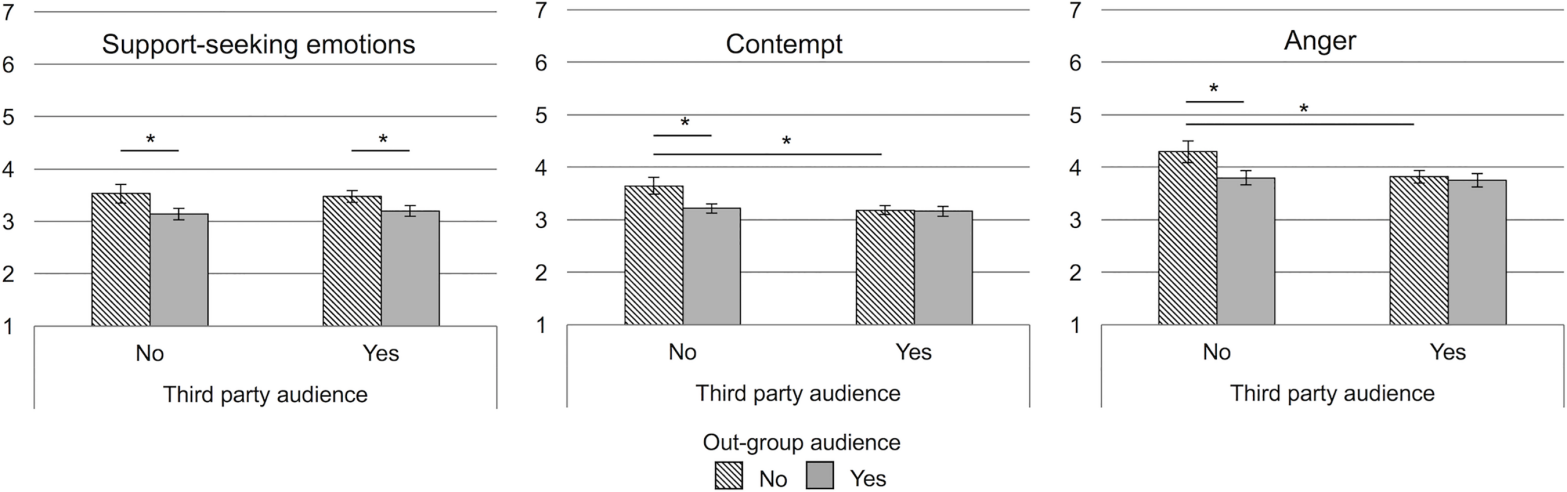
Support-seeking emotions, contempt, and, anger as experienced and expressed towards different audiences (across bogus pipeline conditions) in Study 2. Error bars depict 95% confidence intervals. *p < .05.

Having established confidence in our measures, we focus in the following on the effects of the different audiences on emotions. Results are depicted in Fig 2.

Out-group audience had an effect on support-seeking emotions, *F*(1,79) = 17.09, *p* < .001, η_p_^2^ = .18. Fig 2 shows that, similar to Study 1, participants expressed less support-seeking emotions whenever the out-group was addressed. Third party audience on the other hand did not have an effect on support-seeking emotions, *F*(1,79) = 0.01, *p* = .94, η_p_^2^ < .001 and also the interaction between both audience factors was not significant, *F*(1,79) = 0.85, *p* = 0 .36, η_p_^2^ = .01.

For contempt, we found main effects of both out-group audience, *F*(1,79) = 14.24, *p* = .001, η_p_^2^ = .15, and third party audience, *F*(1,79) = 12.21, *p* = .001, η_p_^2^ = .14 as well as a significant interaction of the two factors, *F*(1,79) = 11.76, *p* = .001, η_p_^2^ = .13. Simple main effects showed that, similar to Study 1, compared to the no audience condition less contempt was expressed towards the out-group audience, *F*(1,79) = 15.97, *p* < .001, η_p_^2^ = .17, and towards the third party, *F*(1,79) = 16.18, *p* < .001, η_p_^2^ = .17. Expression towards both audiences at the same time and towards the separate audiences did not differ significantly, *p*s >.43. This suggests that the expression of contempt was always lower than experience, irrespective of who the audience was.

The pattern of results for anger was similar to that of contempt. Again, we found main effects of out-group audience, *F*(1,79) = 9.28, *p* = .003, η_p_^2^ = .11, and third party audience, *F*(1,79) = 8.20, *p* = .01, η_p_^2^ = .09, as well as a significant interaction between the two factors, *F*(1,79) = 6.60, *p* = .01, η_p_^2^ = .08. Simple main effects comparing anger in the no audience condition to anger expressed towards the out-group, *F*(1,79) = 10.40, *p* = .002, η_p_^2^ = .12, and towards the third party, *F*(1,79) = 10.91, *p* = .001, η_p_^2^ = .12, showed that expression was in both cases lower than experience. At the same time expression towards both audiences did not differ from expression towards separate audiences, *p*s >.43, suggesting that also the expression of anger is overall lower than experience, irrespective of the actual audience.

#### Goals

To assess whether goal importance differed between audiences we computed two 2 (experienced emotions bogus pipeline: no vs. yes) x 2 (expressed emotions bogus pipeline: no vs. yes) x 3 (audience: out-group vs. third party vs. both) mixed ANOVAs. Note that goals were only measured with respect to the two audiences and their combination (i.e., three in all) so we cannot use our 2x2 design for these factors so revert to a single (repeated measures) factor comparing these conditions. If audience showed an effect, planned contrasts (repeated) were computed. Neither bogus pipeline procedures affected goals, *p*s ≥ .17 (only for relationship disruption we found a marginally significant interaction of both factors, *F*(1,77) = 3.38, *p* = .07, η_p_^2^ = .04).

The inclination to call for support differed between audiences, *F*(2,158) = 15.90, *p* < .001, η_p_^2^ = .17, and was, as expected, higher for the third party audience (*M* = 4.34, *SD* = 1.41) than for the out-group audience (*M* = 3.82, *SD* = 1.25), *F*(1,79) = 23.86, *p* < .001, η_p_^2^ = .23, but also than for both audiences at the same time (*M* = 4.14, *SD* = 1.38), *F*(1,79) = 7.44, *p* = .01, η_p_^2^ = .09. These findings confirm our prediction that support would be sought primarily from the third party.

Next, we tested whether the goal to call for support was predicted by expressed support-seeking emotions. We computed three hierarchical multiple regressions (per audience) and entered both bogus pipeline factors, experienced anger, contempt and help-seeing emotions in Step 1, followed by expressed anger, contempt and support-seeking emotions in Step 2.

For each of the audiences, at Step 1 experienced support-seeking emotions were the best predictor for the goal to call for support. This effect however was overridden when we added expressed emotions in Step 2: Here, expressed support-seeking emotions were the only predictor of call for support from each of the audiences (for statistics see Table 4).

**Table 4:** Summary of hierarchical regression analysis for emotions predicting the goal to call for support in Study 2 (*N* = 83).

Audience	Predictor	*B*	*SE B*	β
Out-group	Step 1			
	Bogus Pipeline experience	-0.2	0.25	-.08
	Bogus Pipeline expression	0.03	0.25	.12
	Experienced anger	0.13	0.11	.15
	Experienced contempt	0.16	0.17	.13
	**Experienced support-seeking emotions**	**0.22**	**0.1**	.27*
	Step 2			
	Bogus Pipeline experience	-0.11	0.24	-.05
	Bogus Pipeline expression	-0.11	0.24	-.04
	Experienced anger	-0.002	0.11	-.002
	Experienced contempt	0.09	0.17	.07
	Experienced support-seeking emotions	-0.003	0.13	-.003
	Expressed anger	0.17	0.11	.23
	Expressed contempt	-0.08	0.16	-.07
	**Expressed support-seeking emotions**	**0.36**	**0.13**	.45**
Third Party	Step 1			
	Bogus Pipeline experience	-0.07	0.28	-.03
	Bogus Pipeline expression	-0.35	0.28	-.12
	Experienced anger	0.19	0.11	.20
	Experienced contempt	0.13	0.19	.09
	**Experienced support-seeking emotions**	**0.31**	**0.11**	.33**
	Step 2			
	Bogus Pipeline experience	0.06	0.26	.02
	**Bogus Pipeline expression**	**-0.57**	**0.27**	-.20*
	Experienced anger	0.15	0.12	.15
	Experienced contempt	0.06	0.18	.04
	Experienced support-seeking emotions	-0.04	0.14	-.04
	Expressed anger	0.07	0.13	.08
	Expressed contempt	-0.14	0.2	-.12
	**Expressed support-seeking emotions**	**0.5**	**0.15**	.60**
Both groups	Step 1			
	Bogus Pipeline experience	-0.04	0.28	-.02
	Bogus Pipeline expression	-0.35	0.28	-.13
	Experienced anger	0.17	0.12	.18
	Experienced contempt	0.02	0.19	.01
	**Experienced support-seeking emotions**	**0.33**	**0.11**	.35**
	Step 2			
	Bogus Pipeline 1	0.01	0.27	.004
	Bogus Pipeline 2	-0.5	0.28	-.18
	Experienced anger	0.12	0.13	.13
	Experienced contempt	-0.05	0.19	-.04
	Experienced support-seeking emotions	0.05	0.15	.05
	Expressed anger	0.05	0.14	.05
	Expressed contempt	-0.003	0.19	-.002
	**Expressed support-seeking emotions**	**0.35**	**0.15**	.43*

*Note*. For out-group audience *R*^2^ = .20 for Step 1; Δ*R*^2^ = .12 for Step 2 (*p*s < .05). For third party audience *R*^2^ = .27 for Step 1; Δ*R*^2^ = .13 for Step 2 (*p*s < .05). For both groups audience *R*^2^ = .23 for Step 1; Δ*R*^2^ = .09 for Step 2 (*p*s < .05).

*p < .05.

** p < .01

We expected that it is more important for participants to show that their relationship with Dutch students (i.e., the out-group) is disrupted in communication with them and indeed we found a difference between audiences, *F*(2,154) = 10.14, *p* < .001, η_p_^2^ = .12. To our surprise however communicating relationship disruption was more important to participants in emotion expression towards the third party (*M* = 3.60, *SD* = 1.55) and not the out-group (*M* = 3.01, *SD* = 1.39), *F*(1,77) = 16.85, *p* < .001, η_p_^2^ = .18; The importance of communicating relationship disruption did not differ between third party and both audiences (*M* = 3.41, *SD* = 1.58), *F*(1,77) = 2.57, *p* = .11, η_p_^2^ = .03.

In line with this, showing that the relationship with the out-group is disrupted is only predicted by expressed support-seeking emotions towards the third party and not, as predicted, by expressed contempt towards the out-group (see Table 5). If both audiences were addressed expressed anger was the best predictor. It should be noted however that in both cases in the communication with the third party and both audiences the models were non-significant.

**Table 5:** Summary of hierarchical regression analysis for emotions predicting the goal to distance from the out-group in Study 2 (*N* = 83).

Audience	Predictor	*B*	*SE B*	β
Out-group	*Step 1*			
	Bogus Pipeline experience	0.09	0.31	.03
	Bogus Pipeline expression	0.4	0.31	.15
	Experienced anger	0.19	0.13	.20
	Experienced contempt	-0.09	0.21	-.06
	Experienced support-seeking emotions	0.1	0.12	.11
	*Step 2*			
	Bogus Pipeline experience	0.12	0.3	.04
	Bogus Pipeline expression	0.29	0.3	.11
	Experienced anger	0.01	0.14	.01
	Experienced contempt	-0.15	0.22	-.11
	Experienced support-seeking emotions	0.09	0.17	.10
	Expressed anger	0.32	0.14	.38
	Expressed contempt	-0.19	0.2	-.16
	Expressed support-seeking emotions	0.12	0.16	.13
Third Party	*Step 1*			
	Bogus Pipeline experience	-0.29	0.34	-.09
	Bogus Pipeline expression	-0.11	0.34	-.04
	Experienced anger	0.11	0.14	.10
	Experienced contempt	0.13	0.23	.08
	Experienced support-seeking emotions	0.09	0.14	.08
	*Step 2*			
	Bogus Pipeline experience	-0.17	0.33	-.06
	Bogus Pipeline expression	-0.4	0.34	-.13
	Experienced anger	-0.004	0.16	-.004
	Experienced contempt	0.04	0.23	.02
	Experienced support-seeking emotions	-0.26	0.18	-.25
	Expressed anger	0.19	0.16	.20
	Expressed contempt	-0.15	0.25	-0.12
	**Expressed support-seeking emotions**	**0.48**	**0.19**	.54**
Both groups	*Step 1*			
	Bogus Pipeline experience	-0.11	0.36	-.04
	Bogus Pipeline expression	0.03	0.36	.01
	Experienced anger	0.09	0.15	.08
	Experienced contempt	0.003	0.24	.002
	Experienced support-seeking emotions	0.11	0.15	.10
	*Step 2*			
	Bogus Pipeline 1	-0.11	0.34	-.03
	Bogus Pipeline 2	-0.26	0.35	-.08
	Experienced anger	-0.17	0.17	-.15
	Experienced contempt	-0.05	0.24	-.03
	Experienced support-seeking emotions	-0.09	0.19	-.08
	**Expressed anger**	**0.45**	**0.17**	.47**
	Expressed contempt	-0.1	0.25	-.08
	Expressed support-seeking emotions	0.16	0.2	.17

Note. For out-group audience *R*^2^ = .07 for Step 1; Δ*R*^2^ = .08 for Step 2 (both models ns). For third party audience *R*^2^ = .06 for Step 1; Δ*R*^2^ = .14 for Step 2 (*p* < .05 for Step 2). For both groups audience *R*^2^ = .03 for Step 1; Δ*R*^2^ = .13 for Step 2 (both models ns).

*p < .05.

** *p* < .01

### Discussion

Neither bogus pipeline procedures influenced emotion reports. As emotion reports did not differ between conditions where insincere responding would be possible (i.e., no verification via bogus pipeline) or would be uncovered (i.e., bogus pipeline conditions) this gives us confidence that emotions reported in the no audience condition indeed reflect experienced emotions. Moreover, it also suggests that emotions expressed in the different audience conditions would be similarly expressed in actual confrontations.

We replicated the finding that support-seeking emotions expression towards the out-group is lower than experience while expression of it towards the powerful third party is similar to what they experience. Further, the goal to call for support was more important in the communication with the third party than the out-group. As predicted, emotion expression was used to accomplish this goal: Expressed support-seeking emotions were the strongest predictor of call for support. This was true for all audiences, however it is important to note that both the expression of support-seeking emotions and the goal to call for support were lower for the out-group audience. This finding provides support for the proposed second component of strategic emotion expression, namely that expression has a stronger link to a desired goal than experience. In addition, we found virtually no evidence that the expression of contempt is used to distance from the out-group. Potentially, distancing from the out-group is a less important goal and was already achieved by the out-group when offending the in-group. Also, in the scenario used the out-group does not have any power over the handling of the conflict. This might have made strategy less important here. While we had expected that distancing from the out-group would be particularly important in the communication with the out-group, results showed that it was in fact more important in the communication with the third party or both audiences at the same time. In these conditions, we also find links between emotion expression and distancing from the out-group: Interestingly, we found that expressed support-seeking emotions were used also to communicate distancing from the out-group towards the third party while expressed anger was used in the communication with both audiences. We can only draw careful and tentative conclusions here but it might well be that contempt is perceived as too destructive to be used for any strategic purposes intended to advance the situation for the in-group. Support-seeking emotions and anger on the other hand may have served to create distance between the in-group and the out-group by blaming the out-group for the disadvantages faced.

While the results of Study 2 were largely in line with results of Study 1, we did find some noticeable differences with respect to emotion expression. We will address these in the general discussion.

## General discussion

In two studies, we investigated whether members of disadvantaged groups express emotions strategically in order to tackle their situation. To do so, we looked at two components of their emotion expression: Firstly, we tested whether emotion expression differs depending on the audience rather than reflecting experience. In both studies, we found that support-seeking emotion expression towards the out-group was played down in comparison to emotion experience (i.e., no audience). We further found less expression of contempt in comparison to experience. In Study 1 this was only true in the case of the third party audience but in Study 2 we found an overall reduction of contempt expression. With respect to anger we found mixed results. While in Study 1 we found no differences between experience and expression towards out-group or third party in Study 2 we found an overall reduction in expression compared to experience. As such, we found support for the claim that expression may differ from experience for different emotions.

Secondly, in Study 2 we looked at the association between emotions and goals, predicting that members of disadvantaged groups would use support-seeking emotion expression to call for support and contempt expression to distance from the out-group. We indeed found that expressed support-seeking emotions predicted calling for support over and above experienced support-seeking emotions. However, we did not find the expected link between contempt and distancing. Instead, we found an association between expressed support-seeking emotions and distancing when the third party was the only audience and an association between expressed anger and distancing when communicating with both audiences at the same time. Thus, rather than using contempt to distance from the out-group, we have some evidence that participants used anger to do so. We further interpret the association between support-seeking emotions and distancing from the out-group in front of the third party audience as a way to blame the out-group for the in-group’s disadvantage. The motivation for this may be to create distance between the third party and the out-group, which would serve the in-group’s interests.

Overall, these findings support the proposed association between expressed emotions and goals, but our results suggest that this may be particularly true for beneficial goals (e.g., enlisting support) and less so for destructive goals in the communication with the out-group (e.g., distancing from the out-group). Arguably, pursuing destructive goals in the communication with an out-group requires less strategy while beneficial goals on the other hand–especially in conflicts–may require more strategic considerations and adjustments to emotion expression. Nonetheless we cannot rule out that attack-related emotions such as anger, contempt, or even hatred are never expressed in a strategic manner. In fact, looking at actual conflicts such as the Israeli-Palestinian conflict, it seems likely that such emotions could indeed be expressed strategically to provoke retaliatory responses, which call (international) attention to the conflict.

These findings advance our understanding of emotion expression in intergroup conflicts in two important ways. The function of expressed emotions has so far mostly been investigated with a focus on how expressed emotions influence an audience [4,5,9]. By showing that emotions can be expressed strategically we complement those findings from the expresser perspective. Not only do emotions influence an audience it seems as if expressers may specifically intend such influence, which is an important link in the inference of strategic behavior. In particular, our findings complement those of Kamans et al. [4]. While they found that third parties were particularly likely to support a disadvantaged group that expressed fear, we could show that members of disadvantaged groups used fear (together with sadness) to enlist third party support. Thus it seems as if participants were–at least to a certain extent–aware that the emotions they communicated to an audience would influence the audience. This suggests that lay theories that we hold regarding the effects of support-seeking emotions match with research findings: Not only do support-seeking emotions enlist support but they are also consciously used to do so.

The fact that we found a reduction of contempt expression (towards the third party in Study 1 and all audiences in Study 2) suggests that participants were aware of its potential detrimental effects [5] however we were not able to establish the link between contempt and the distancing goal. This could either mean that the distancing function is not part of people’s lay beliefs regarding the effects of contempt or that participants simply did not want to use contempt to distance. Contempt and distancing occur mostly in situation with repeated frustration or unfair treatment [12,26], thus the fact that we used a scenario with a single unfair incident may not have been sufficient to trigger the use of contempt to distance. For anger, the results were mixed and importantly we did not find an association between anger expression and beneficial goals. On the contrary, we found an association between expressed anger and distancing from the out-group when communicating with both audiences. This suggests that the positive effects of anger expression that have been demonstrated [5, 27] are not incorporated in lay beliefs. This fits well with the common lay understanding of anger as a negative emotion, despite the apparently positive effects it can have [27].

Further our results also provide interesting insights in light of instrumental emotion regulation. While research in this area mostly focuses on how individuals want to feel [14,15,16] we could show that also what individuals want to express for utilitarian purposes.

A question often raised in emotion research is how accurately we can measure emotions and while self-reports seen to be useful measures in general they do have limitations [28]. By employing two different experimental designs (i.e., a between-subjects design in Study 1 and a within-subjects design in Study 2) while keeping the context constant in both studies we aimed to reduce measurement error. Using both designs we found a reduction of support-seeking expression towards the out-group on comparison to experience. To further verify the results obtained using rating scales we used the bogus pipeline technique in Study 2. The fact that we did not find any differences in emotion reports with and without the bogus pipeline manipulations gives us some confidence in the measure used (Study 2). Nevertheless, it would be desirable to replicate the differences found between emotion experience and expression also with other measures. Importantly, such measures have to distinguish between distinct emotions so that many physiological measures would not be appropriate [29]. Such measures may however be useful to uncover how emotion expression is strategically adjusted. For example, given that we mainly observed down-regulation of emotion expression it may be worth exploring whether this is achieved through antecedent- or response-focused appraisal where the latter should be accompanied by stronger physiological reactions [30]. Further, any written or verbal account should be considered in future research.

### Limitations and suggestions for future research

We would like to point out some limitations that could give rise to follow-up research. Firstly, while our results support the central assumption that emotion expression may differ from emotion experience (i.e., the first component of strategic emotion expression) it is necessary to discuss some differences in the overall result patterns of Study 1 and Study 2. For support-seeking emotions, contempt, and anger we find slightly different patterns in the two studies that, taken together, suggest that different strategies may have been used. It seems as if participants in Study 1 aimed to maximize their chances of improving the situation while participants in Study 2 appeared somewhat more selective and careful: For example, expression of support-seeking emotions towards both audiences at the same time resembled experience in Study 1 but were lower in Study 2. Though conclusions should be drawn carefully, this suggests that participants in Study 1 sought every opportunity to communicate their need for support (and thus even took the risk to appear weak in front of the out-group as long if the third party was addressed as well) but avoided this risk in Study 2. These two different strategies might also explain the different patterns we found for anger. Willingness to seek every opportunity to improve the situation could have motivated the expression of anger while higher cautiousness in Study 2 motivated the generally reduced expression. As the conflict used in the two studies was identical it seems likely that these differences may have been the result of differences in the design. While participants in Study 1 only got to communicate their emotions once (or never in the no audience condition), they may have been tried to optimize the outcome for the in-group. In Study 2 on the other hand participants got to communicate with all audiences which allowed them to be more selective.

Secondly, with respect to strategic emotion expression in general, we have initial evidence that participants used their emotions as a subtle tool to influence the audience. In order to further strengthen the claim that participants choose expressed emotions due to their subtlety future research should compare emotion expression as a way to communicate with and influence an audience to more direct ways of communication (such as language and action). For example, in ongoing research we currently investigate both direct verbal and subtle emotional calls for support.

Thirdly, the finding that support-seeking emotions are used to enlist support should be tested under less restricted conditions. In the studies presented here we tested our hypotheses only in one particular, artificial context with a powerful and undecided third party. While this allowed us to explore the fundamentals of strategic emotion expression in follow-up research we have turned to investigate whether the emotional call for support is primarily driven by the fact that the third party had power or whether that it was not primarily responsible for the proposed changes that would bring about disadvantages for the in-group.

## Conclusion

Our research contributes to the understanding of the function of emotion in intergroup conflicts in general and in particular to the role of emotion expression. While the function of emotions was mostly studied in the context of how experienced emotions influence own actions [3] and how expressed emotions influence actions of audiences [4,5,31] we can now add that emotion expression itself is also likely to serve a function, namely to pursue a goal that is considered beneficial for the own group. Thus, not only experienced group-based emotions are regulated for instrumental purposes [16] but also expressed emotions. The notion of benefit seems to be important as we did not find strategic emotion expression of potentially destructive emotions towards the out-group. Thus, emotion expression is more than merely expressing what we feel but serves as a tool to overcome a disadvantaged situation.

## Supporting information

S1 File Additional measures. This file contains short descriptions and summaries of the results of additional measures.
(DOCX)

S2 File Study 1 moderation analyses. This file contains a summary of exploratory analyses of identification as a moderator.
(DOCX)

S3 File Study material. This file contains the exact wording of the study material.
(DOCX)

S1 Table Study 1 and 2 descriptive statistics of additional emotions. (PDF)

S1 Fig Study 2 scatterplots calling for support and all significant predictors. (PDF)

S2 Fig Study 2 scatterplots out-group distancing and all significant predictors. (PDF)

## References

[1] Lindner EG. Emotion and conflict: Why it is important to understand how emotions affect conflict and how conflict affects emotions In: Deutsch M, Coleman PT, Marcus EC, editors. The handbook of conflict resolution: Theory and practice: Jossey-Bass San Francisco, CA; 2006 p. 268–293.

[2] van Zomeren M, Spears R, Fischer AH, Leach CW. Put Your Money Where Your Mouth Is! Explaining Collective Action Tendencies Through Group-Based Anger and Group Efficacy. J Pers Soc Psychol 2004 11;87(5):649–664. 10.1037/0022-3514.87.5.649 15535777

[3] Mackie DM, Devos T, Smith ER. Intergroup emotions: Explaining offensive action tendencies in an intergroup context. J Pers Soc Psychol 2000 10;79(4):602–616. 11045741

[4] Kamans E, van Zomeren M, Gordijn EH, Postmes T. Communicating the right emotion makes violence seem less wrong: Power-congruent emotions lead outsiders to legitimize violence of powerless and powerful groups in intractable conflict. Group Processes & Intergroup Relations 2014 05;17(3):286–305.

[5] de Vos B, van Zomeren M, Gordijn EH, Postmes T. The communication of 'pure' group-based anger reduces tendencies toward intergroup conflict because it increases out-group empathy. Person Soc Psychol Bull 2013 08;39(8):1043–1052. 10.1177/0146167213489140 23709041

[6] Ellsworth PC, Scherer KR. Appraisal processes in emotion In: Davidson RJ, Scherer KR, Goldsmith HH, editors. Handbook of affective sciences New York, NY, US: Oxford University Press; 2003 p. 572–595.

[7] Smith ER, Seger CR, Mackie DM. Can emotions be truly group level? Evidence regarding four conceptual criteria. J Pers Soc Psychol 2007 09;93(3):431–446. 10.1037/0022-3514.93.3.431 17723058

[8] Clark MS, Pataki SP, Carver VH. Some thoughts and findings on self-presentation of emotions in relationships In: Fletcher GJO, Fitness J, editors. Hillsdale, NJ, England: Lawrence Erlbaum Associates, Inc; 1996 p. 247–274.

[9] Van Kleef GA. How emotions regulate social life: The emotions as social information (EASI) model. Current Directions in Psychological Science 2009 06;18(3):184–188.

[10] Van Kleef GA, Van Doorn EA, Heerdink MW, Koning LF. Emotion is for influence. European Review of Social Psychology 2011 01;22(1):114–163.

[11] Kamans E, Otten S, Gordijn EH. Power and threat in intergroup conflict: How emotional and behavioral responses depend on amount and content of threat. Group Processes & Intergroup Relations 2011 05;14(3):293–310.

[12] Fischer AH, Roseman IJ. Beat them or ban them: The characteristics and social functions of anger and contempt. J Pers Soc Psychol 2007 07;93(1):103–115. 10.1037/0022-3514.93.1.103 17605592

[13] Xiao E., Houser D. Emotion expression in human punishment behavior. Proceedings of the National Academy of Sciences of the United States of America 2005;102(20):7398–401. 10.1073/pnas.0502399102 15878990 PMC1129129

[14] Tamir M. What do people want to feel and why?: Pleasure and utility in emotion regulation. Current Directions in Psychological Science 2009 04;18(2):101–105.

[15] Tamir M, Mitchell C, Gross JJ. Hedonic and instrumental motives in anger regulation. Psychological Science 2008 04;19(4):324–328. 10.1111/j.1467-9280.2008.02088.x 18399883

[16] Goldenberg A, Halperin E, van Zomeren M, Gross JJ. The process model of group-based emotion: Integrating intergroup emotion and emotion regulation perspectives. Personality and Social Psychology Review 2016 05;20(2):118–141. 10.1177/1088868315581263 25870386

[17] Zummuner VL, Fischer AH. The social regulation of emotions in jealousy situations: A comparison between Italy and the Netherlands. Journal of Cross-Cultural Psychology 1995 03;26(2):189–208.

[18] Fischer AH, Manstead ASR, Evers C, Timmers M, Valk G. Motives and norms underlying emotion regulation In: Philippot P, Feldman RS, editors. Mahwah, NJ, US: Lawrence Erlbaum Associates Publishers; 2004 p. 187–210.

[19] Ekman P, Friesen WV. The repertoire of nonverbal behavior: Categories, origins, usage, and coding. Semiotica 1969 01; (1):49–98.

[20] Andrade EB, Ho T. Gaming emotions in social interactions. Journal of Consumer Research 2009 12;36(4):539–552.

[21] Leach CW, van Zomeren M, Zebel S, Vliek MLW, Pennekamp SF, Doosje B, et al Group-level self-definition and self-investment: A hierarchical (multicomponent) model of in-group identification. J Pers Soc Psychol 2008 07;95(1):144–165. 10.1037/0022-3514.95.1.144 18605857

[22] Tausch N, Becker JC, Spears R, Christ O, Saab R, Singh P, et al Explaining radical group behavior: Developing emotion and efficacy routes to normative and nonnormative collective action. J Pers Soc Psychol 2011 07;101(1):129–148. 10.1037/a0022728 21500925

[23] Jones EE, Sigall H. The bogus pipeline: A new paradigm for measuring affect and attitude. Psychol Bull 1971 11;76(5):349–364.

[24] Faul F, Erdfelder E, Lang A, Buchner A. G* Power 3: A flexible statistical power analysis program for the social, behavioral, and biomedical sciences. Behavior research methods 2007;39(2):175–191. 17695343 10.3758/bf03193146

[25] Postmes T, Haslam SA, Jans L. A single-item measure of social identification: Reliability, validity, and utility. British Journal of Social Psychology 2013 12;52(4):597–617. 10.1111/bjso.12006 23121468

[26] Fischer A, Giner-Sorolla R. Contempt: Derogating others while keeping calm. Emotion Review 2016;8(4):346–357.

[27] Hess U. Anger is a positive emotion In: Parrott WG, editor. New York, NY, US: Guilford Press; 2014 p. 55–75.

[28] Robinson MD, Clore GL. Belief and feeling: evidence for an accessibility model of emotional self-report. Psychol Bull 2002;128(6):934 12405138 10.1037/0033-2909.128.6.934

[29] Maus IB, Robinson MD. Measures of emotion: A review. Cognition and emotion. 2009 2;23(2):209–237. 10.1080/02699930802204677 19809584 PMC2756702

[30] Gross JJ. Antecedent-and response-focused emotion regulation: Divergent consequences for experience, expression, and phyiology. Journal of personality and social psychology. 1998 1;74(1):224–237. 9457784 10.1037//0022-3514.74.1.224

[31] de Vos B, van Zomeren M, Gordijn EH, Postmes T. When does the communication of group-based anger increase outgroup empathy in intergroup conflict? The role of perceived procedural unfairness and outgroup consensus. Group Processes & Intergroup Relations 2016:Forthcoming.

