# Supplementary material for: When to reveal what you feel: How emotions towards antagonistic out-group and third party audiences are expressed strategically
Source: PLoS One. 2018 Sep 7;13(9):e0202163. doi: 10.1371/journal.pone.0202163 (PMC6128462; doi:10.1371/journal.pone.0202163)
Supplement: S1 File — This file contains short descriptions and summaries of the results of additional measures. (DOCX) [file pone.0202163.s001.docx]

In the following, we present an overview over measures which were not central to answer our research questions but were assessed for explorative reasons. We describe each measure and list central findings. Further analyses and the data are available upon request.

**Additional Emotions**

In each study, we measured three additional negative emotions (disappointment, dejection, agitation) and three positive emotions (hope, sympathy, happiness) along with the key emotions. Here, we only present audience effects on each emotion and focus on the comparison between no audience conditions (experience) and single audience expression conditions. Descriptive statistics can be found in S1 Table.

In Study 1, we found an interaction effect of audiences on dejection, *F*(1,82) = 7.76, *p* = .01, η_p_^2^ = 0.09, disappointment, *F*(1,82) = 6.80, *p* = .01, η_p_^2^ = 0.08, and agitation, *F*(1,82) = 5.14, *p* = .03, η_p_^2^ = 0.06. For all three negative emotions simple effects comparing either audience with the baseline conditions were non-significant. For positive emotions, we did not find any effects of audiences, all *p*s > .05.

In Study 2, we founds effects of audience on dejection, *F*(3,237) = 6.27, *p* = .001, η_p_^2^ = .07 (experience vs. out-group audience: F(1,79) = 6.01, p = .02, η_p_^2^ = .07) , agitation, *F*(3,237) = 5.69, *p* = .003, η_p_^2^ = .07 (experience vs. out-group audience: *F*(1,79) = 9.72, p = .003, η_p_^2^ = .11), and sympathy, *F*(3,237) = 3.90, *p* = .013, η_p_^2^ = .05 (experience vs. out-group audience: *F*(1,79) = 2.91, *p* = .09, η_p_^2^ = .04). Audience did not have an effect on disappointment, hope, and happiness, all *p*s > .05.

**Broader goals**

In Study 2, we embedded the focal goals (calling for support and stressing relationship disruption) in several other goals of emotion expression to obscure the focal goals and analyzed the results for explorative reasons. Irrespective of audience it was of little importance to participants to express emotions as expected by the audience (*M* = 2.73, *SD* = 1.31) and to show outrage (*M* = .3.43, *SD* = 1.49). It was however important to participants to appear reasonable (*M* = 5.56, *SD* = 1.01). Only with respect to improving the in-group’s position we found an effect of audience, *F*(2, 156) = 19.67, *p* < .001, η_p_^2^ = .20. Improving the in-group’s position was more important when participants expressed their emotions towards the third party (*M* = 5.94, *SD* = 0.99) than towards the out-group (*M* = 5.37, *SD* = 1.27), *F*(1,78) = 27.26, *p* < .001, η_p_^2^ = .25, or both groups (*M* = 5.82, *SD* = 0.98), *F*(1,78) = 3.54, *p* = .06, η_p_^2^ = .04.

**Expected reaction from audience**

In Study 2, we assessed expected reactions from each audience. Participants indicated to what extent they expected an audience to distance from the in-group and to support the in-group in response to emotion expression. Results were in line with goals, i.e., participants called for support from the third party but not the out-group and in line expected support from the third party and distancing from the out-group.

**Action intentions**

In Study 1, we used different measures to assess action intentions. These measures were taken after emotion expression. Participants read various statements, allegedly written by fellow students, and indicated how much they would support each statement. Statements expressed either destructive actions, constructive actions, experience of powerlessness, approval of the proposed changes, disinterest in the topic (four statements per category). We found virtually no differences for the willingness to engage in various actions when facing different audiences which suggests that – other than for support-seeking emotions - no different behavioral strategies are favored over others for different audiences.

**Likelihood of influence**

Perceived likelihood of influence was measured after expressed emotions (Study 1) or after goals had been measured (Study 2). Participants rated to what extent an audience would be influenced by their own (individual) emotions and by the emotions of their group. Overall, collective emotions were perceived to be more likely to influence audiences than individual emotions.

**Procedural fairness**

This was measured at the end of both studies with four items, namely *(International/ Dutch) students have a say/are unheard in the decision making process, the decision of the university to let a committee decide about the proposal is fair, the handling of the proposal is unjust.* In both studies, procedural fairness was perceived as being low.
