# Supplementary material for: When to reveal what you feel: How emotions towards antagonistic out-group and third party audiences are expressed strategically
Source: PLoS One. 2018 Sep 7;13(9):e0202163. doi: 10.1371/journal.pone.0202163 (PMC6128462; doi:10.1371/journal.pone.0202163)
Supplement: S2 File — This file contains a summary of exploratory analyses of identification as a moderator. (DOCX) [file pone.0202163.s002.docx]

**Moderation Effect of Identification**

For Study 1, we tested whether identification moderates the effects of audience on emotions. While we thought it was likely that identification influences strategic emotion expression we were unsure about the direction of the effect: Arguably, strategy could be more important for high identifiers (i.e., differences between experienced and expressed emotions should be more pronounced). At the same time, as high identifiers often experience group-based emotions more strongly, it is possible that high identifiers are less strategic but are guided by their strongly felt emotions. We tested the moderation effect of identification on support-seeking emotions, contempt, and anger by using the SPSS Process Macro (Model 3). For none of the three emotions did we find a significant three-way interaction yet we did find interactions between identification and out-group audience for contempt and anger. To follow up on these effects we performed two additional moderation analyses (Model 1, controlling for third party audience). For anger, *B*= -0.87, *SE* = .37, *t*(81) = -2.38, *p* = .02, we found that low identifiers expressed more anger when the out-group was present than when it was not, *B* = .84, *SE* = .42, *t*(81) = 2.01, *p* = .047. The pattern for contempt was similar, *B* = -0.75, SE = .30, *t*(.81) = -2.55, *p* = .01. Low identifiers expressed more contempt when the out-group was present than when it was not, *B* = 0.94, *SE* = .34, *t*(81) = .2.73, *p* = .01.

This suggests that particularly low identifiers were influenced by the presence of the out-group when it came to the expression of anger and contempt while for expression for high identifiers was not.
