# Supplementary material for: When to reveal what you feel: How emotions towards antagonistic out-group and third party audiences are expressed strategically
Source: PLoS One. 2018 Sep 7;13(9):e0202163. doi: 10.1371/journal.pone.0202163 (PMC6128462; doi:10.1371/journal.pone.0202163)
Supplement: S3 File — This file contains the exact wording of the study material. (DOCX) [file pone.0202163.s003.docx]

**Survey questions**

Here, we report the exact wording used in Studies 1 and 2 to assess the dependent measures reported in the paper.

***Study 1***

*Appraisals*

Meausred on a scale from 1 (strongly disagree) to 7 (strongly agree)

The proposal is unfair/ The proposal is socially unjust/ The proposal is legitimate/ The proposal is justified.

The proposal is immoral/ The proposal is ethical/ The proposal is unprincipled/ The proposal is morally acceptable.

The proposal renders me uncertain about my future/ Despite of the proposal I still feel secure about the progress of my studies/ The proposal raises concerns about my professional future/ Despite of the proposal I feel that my future is secure.

The proposal came as a complete surprise/ The proposal was to be expected/ The proposal was predictable/ The proposal was made against expectation

The proposal is beyond our control/ The proposal is can only be changed by others/ The proposal is controllable by us/ The proposal is modifiable by us.

*Emotions*

Introduction:

Dutch students made the proposal for additional tuition fees solely for international students. How do you as an international student feel about the proposal and its consequences?

*No audience condition:*

Remember that the data collected in this survey are collected for scientific reasons and will be held confidentially. Neither members of the committee nor Dutch students will have access to the data. Thus, you can express exactly how you feel about the proposal.

*Out-group audience condition*

Remember that the results of this survey will be communicated to the Dutch students. After they explained the motives for their proposal, international students deserve the chance to reply and present their thoughts and feelings to Dutch students. Remember, that members of the committee will not have access to the results of the survey.

*Third party audience condition*

Remember that the results of this survey will be communicated to members of the committee before the committee meets. We are of the opinion, that it is helpful for members of the committee to know about the thoughts and feelings of international students before they make their decision. Remember, that Dutch students will not have access to the result of the survey.

*Both audiences condition*

Remember that the results of this survey will be communicated to Dutch students and members of the committee before the committee meets. We are of the opinion, that it is helpful for members of the committee to know about the thoughts and feelings of international students (as well as the Dutch students), before they make their decision. In addition, we are of the opinion, that after Dutch students explained the motives for their proposal, international students deserve the chance reply and present their thoughts and feelings to Dutch students.

Measured on a scale from 1 (not at all) to 7 (a lot)

I am…

angry/ irritated/ revolted

Sad/ depressed/ down/ scared/ anxious/ frightened

Contemptuous/ scornful/ disdainful

***Study 2***

*Appraisals as in Study 1*

*Emotions and goals measure*

Emotions measured on a scale from 1(not at all) to 7 (a lot)

Goals measured on a scale from 1 (strongly disagree) to 7 (strongly agree)

*Phase 1: No audience condition*

*Bogus pipeline experienced emotions manipulation*

(on and off) We would now like to get to know your thoughts and feelings on a sensitive topic, which we consider is of high importance for you as an international student. As we are going to ask you some questions about the text later please read it carefully. (only on) In order to be able to validate your responses regarding your feelings we will now use the four sensors attached to your face. Before you start reading the text on the next page, please read this information about the Facial Response Sensors:

Facial Response Sensors

When experiencing a certain emotion a specific activity pattern can be observed in facial muscles that is distinct from the pattern of any other emotion. We can control our facial display to a certain degree but the underlying minimal muscular activity is not willfully controllable. Facial Response Sensors can measure this minimal muscular activity so that the specific patterns and thus the experienced emotions can be identified even if one tries to suppress sincerely felt emotions and facial display. In order to measure truly experienced emotions we are going to use four sensors that are attached to your skin with the help of adhesive foil. This method is not dangerous in any way: The sensors simply measure electric potential generated by the muscles. Said differently we only measure electricity that exists in the body anyway and we do not induce electricity. As we cannot control this minimal muscular activity there is no point in reporting emotions insincerely. The use of Facial Response Sensors allows us to cross validate your responses.

Please sit comfortably and try not to move your head while recording. By clicking ">>" the recording will start automatically and will be indicated by a red dot in the upper right corner. We will inform you when the recording stops.

How do you as an international student feel about the proposal and its consequences?

I feel angry/ irritated

I feel depressed/ down/ scared/ frightened

I feel scornful/ disdainful

Phase 2: Expression

*Bogus pipeline expressed emotions manipulation*

(on and off) Imagine that the results of this survey would not be held confidentially but instead would be communicated to Dutch students or staff members before the committee meets: To what extend would you express your emotions in these situations? You might express more or less or the same degree of the emotions as before - this is up to you but we are interested in your response when communicating the emotions to this group. Again there are no right or wrong answers to this question but it is important that you indicate sincerely which emotions you would like to express in this situation. (only on) To be able to validate whether you response sincerely, we now take physiological measurements with the help of the single sensor attached to your hand. Please read the information about it carefully:

Deviation Polygraph

Concealing one’s actual intentions and responding insincerely is accompanied by an increase of arousal. Altered arousal elicits a change in skin conductance response (SCR). This is not willfully controllable and can be measured with the help of a single sensor attached to the back of the hand, commonly the left hand. Of course the level of arousal is also influenced by other factors such as the participation in a study itself. We therefore measure your SCR constantly from now on to assess your mean level of SCR. An increase of arousal due to concealing will stand out of this SCR profile. This method is not dangerous in any way: A change in SCR is a natural response of the body which we simply record. As this reaction cannot be controlled willfully there is no point in concealing your intended expression and answering insincerely. The use of the Deviation Polygraph allows us to cross validate your responses.

Please place your left hand on the marks on the table and try to avoid any movements of your left hand while recording. By clicking ">>" the recording will start automatically and will be indicated by a red dot in the upper right corner. We will inform you when the recording stops.

*Out-group audience condition:*

Please imagine that...

...the results of this survey would be communicated to the Dutch students but not to staff members before the committee meets. After they explained the motives for their proposal, international students deserve the chance to reply and present their thoughts and feelings to Dutch students. To what extend would you express your emotions when taking into account that you were communicating to Dutch students? You might express more or less or the same degree of the emotions as before - this is up to you but we are interested in your response when communicating the emotions to this group.

Again there are no right or wrong answers to this question but it is important that you indicate sincerely which emotion you would like to express in this situation. Remember that we are currently recording your SCR-profile with the Deviation Polygraph in order to be able to validate your responses. Please note that as a change in the SCR profile is a natural reaction to concealing one's actual intentions and responding insincerely there is no point in doing so.

Emotions:

To what extend would you express the following emotions taking into account that you were communicating to Dutch students?

If Dutch students were to hear about our reactions, I would express feeling angry/ irritated

If Dutch students were to hear about our reactions, I would express feeling depressed/ down/ scared/ frightened

If Dutch students were to hear about our reactions, I would express feeling scornful/ disdainful

Goals:

Do you have certain intentions when expressing your emotions to Dutch students?

My intention is to show that we need assistance. / My intention is to show that we are victims.

My intention is to show that our relationship with Dutch students is disrupted.

*Third party audience condition:*

Now we would like you to imagine that...

...the results of this survey will be communicated to the staff members before the committee meets but not to Dutch students. It would be helpful for members of the committee to know about the thoughts and feelings of international students as one of the affected parties before they make their decision. To what extent would you express your emotions when taking into account that you were communicating to staff members before the committee meets? You might express more or less or the same degree of the emotions as before - this is up to you but we are interested in your response when communicating the emotions to this group.

Again there are no right or wrong answers to this question but it is important that you indicate sincerely which emotion you would like to express in this situation. Remember that we are currently recording your SCR-profile with the Deviation Polygraph in order to be able to validate your responses. Please note that as a change in the SCR profile is a natural reaction to concealing one's actual intentions and responding insincerely there is no point in doing so.

Emotions:

To what extend would you express the following emotions taking into account that you were communicating to staff members before the committee meets?

If staff members were to hear about our reactions, I would express feeling angry/ irritated

If staff members were to hear about our reactions, I would express feeling depressed/ down/ scared/ frightened

If staff members were to hear about our reactions, I would express feeling scornful/ disdainful

Goals:

Do you have certain intentions when expressing your emotions to staff members?

My intention is to show that we need assistance. / My intention is to show that we are victims.

My intention is to show that our relationship with Dutch students is disrupted.

*Both audiences condition:*

Now please imagine that...

...the results of this survey will be communicated to Dutch students and staff members before the committee meets. In other words: Here the results will be communicated to both groups, Dutch students and staff members, at the same time. To what extent would you express the following emotions taking into account that you were communicating to both groups, Dutch students and staff members, at the same time? You might express more or less or the same degree of the emotions as before - this is up to you but we are interested in your response when communicating the emotions to this group.

Again there are no right or wrong answers to this question but it is important that you indicate sincerely which emotion you would like to express in this situation. Remember that we are currently recording your SCR-profile with the Deviation Polygraph in order to be able to validate your responses. Please note that as a change in the SCR profile is a natural reaction to concealing one's actual intentions and responding insincerely there is no point in doing so.

Emotions:

To what extend would you express the following emotions taking into account that you were communicating to both groups, Dutch students and staff members, at the same time?

If both groups were to hear about our reactions, I would express feeling angry/ irritated

If both groups were to hear about our reactions, I would express feeling depressed/ down/ scared/ frightened

If both groups were to hear about our reactions, I would express feeling scornful/ disdainful

Goals:

Do you have certain intentions when expressing your emotions to Dutch students and staff members at the same time?

My intention is to show that we need assistance. / My intention is to show that we are victims.

My intention is to show that our relationship with Dutch students is disrupted.
