# Supplementary material for: When to reveal what you feel: How emotions towards antagonistic out-group and third party audiences are expressed strategically
Source: PLoS One. 2018 Sep 7;13(9):e0202163. doi: 10.1371/journal.pone.0202163 (PMC6128462; doi:10.1371/journal.pone.0202163)
Supplement: S1 Table — (PDF) [file pone.0202163.s004.pdf]

S1 Table. Means (*M*), standard deviations (*SD*), and effects of audience (i.e., differences between experience and expression towards a single audience) for all exploratively measured emotions.

		Audience			
		Baseline	Out-group	Third party	Both
Emotion	Study	*M* (*SD*)	*M* (*SD*)	*M* (*SD*)	*M* (*SD*)
Disappointment	1	4.60 (1.25)	3.98 (1.61)	4.08 (1.68)	5.07 (1.11)
	2	4.45 (1.38)	4.25 (1.60)	4.48 (1.64)	4.26 (1.73)
Dejection	1	4.53 (1.23)	3.68 (1.49)	3.85 (1.64)	4.65 (1.08)
	2	4.04 (1.26)	3.75* (1.42)	4.18 (1.53)	3.95 (1.45)
Agitation	1	4.55 (1.16)	3.92 (1.65)	4.03 (1.61)	4.79 (1.20)
	2	4.17 (1.49)	3.70* (1.56)	4.04 (1.69)	3.78 (1.69)
Hope	1	3.78 (1.29)	4.00 (1.20)	3.92 (0.83)	3.65 (1.23)
	2	3.37 (1.25)	3.31 (1.43)	3.20 (1.47)	3.25 (1.53)
Sympathy	1	3.08 (1.16)	3.40 (1.16)	3.03 (1.07)	3.19 (1.37)
	2	3.74 (1.24)	3.95 (1.27)	3.82 (1.24)	4.09 (1.29)
Happiness	1	2.58 (1.37)	3.00 (1.22)	2.83 (1.14)	2.41 (1.17)
	2	2.10 (1.03)	2.16 (1.22)	2.05 (1.21)	2.10 (1.27)

Note. Asterisks indicate significant differences from Baseline. $p < .05$
