# Supplementary figures and images for: When to reveal what you feel: How emotions towards antagonistic out-group and third party audiences are expressed strategically
Source: PLoS One. 2018 Sep 7;13(9):e0202163. doi: 10.1371/journal.pone.0202163 (PMC6128462; doi:10.1371/journal.pone.0202163)

A1

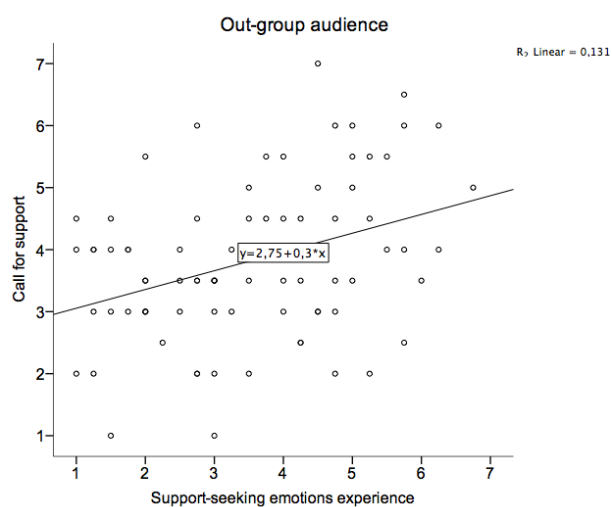

A2

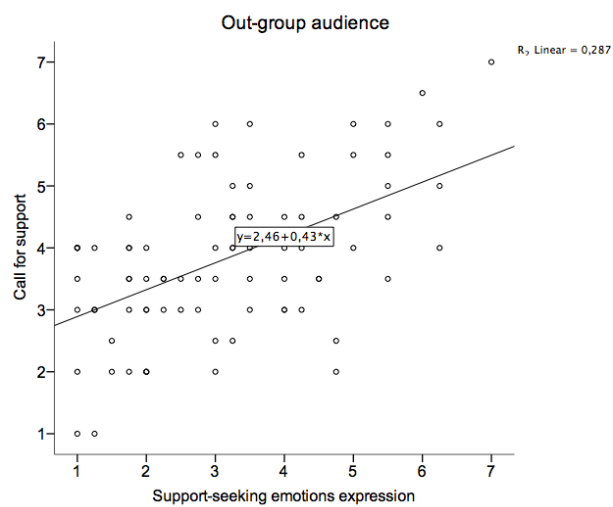

B1

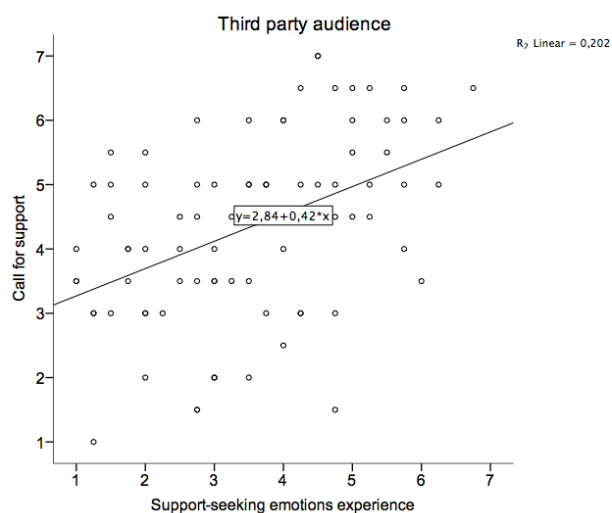

B2

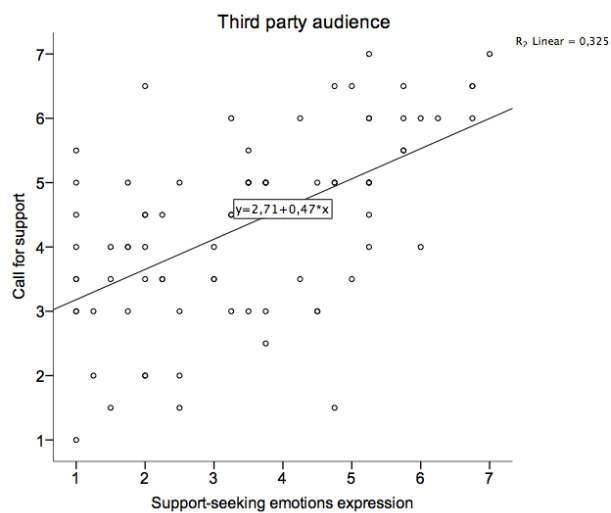

C

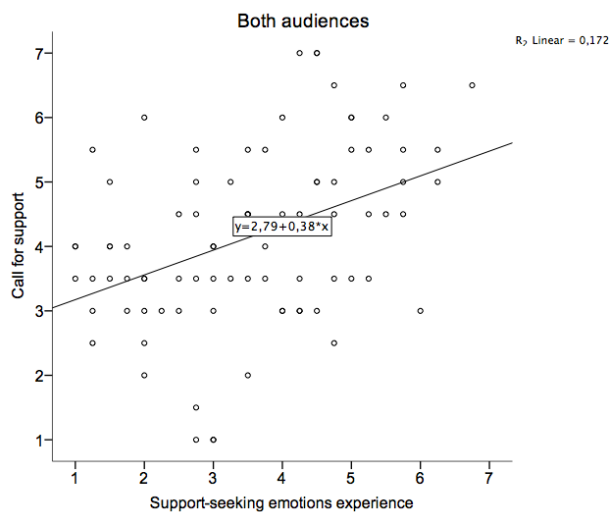

Supplement: S1 Fig — (PDF) [file pone.0202163.s005.pdf]

A

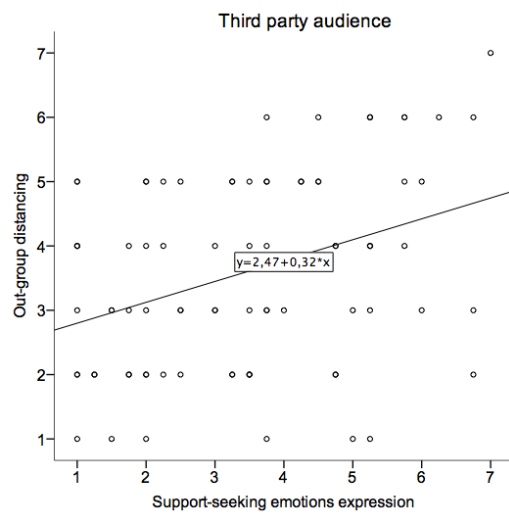

B

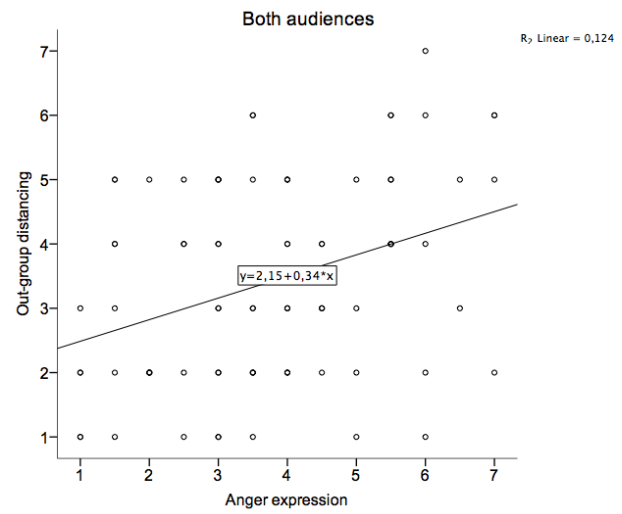

Supplement: S2 Fig — (PDF) [file pone.0202163.s006.pdf]
